# Supplementary material for: Efficacy of Western, Eastern, and Venezuelan Equine Encephalitis (WEVEE) Virus-Replicon Particle (VRP) Vaccine against WEEV in a Non-Human Primate Animal Model
Source: Viruses. 2022 Jul 8;14(7):1502. doi: 10.3390/v14071502 (PMC9321360; doi:10.3390/v14071502)
Supplement: Supplementary file 1 [file viruses-14-01502-s001.zip › viruses-1751419-supplementary.pdf]

Supplementary Materials for

Burke, C.W. et al. Efficacy of Western, Eastern, and Venezuelan Equine Encephalitis (WEVEE) Virus-Replicon Particle (VRP) Vaccine against WEEV in a Non-human Primate Animal Model

Table S1. Summary of Significant Histologic Results by Study Animal

Study ID	Central Nervous System Lesions	Tissues with Lymphoid Hyperplasia	Lesions Not caused by WEEV Infection	Tissues Not Evaluated Histologically
Intramuscular Delivery of WEVEE VRP Vaccine (IMVRP) or Control (IMCTRL)				
IMVRP-1	NSL	Spleen, tonsils, TB LN	NSL	N/A
IMVRP-2	Perivascular cuffs	Spleen, tonsils, LNs	NSL	N/A
IMVRP-3	NSL	Spleen, tonsils, LNs	NSL	N/A
IMVRP-4	NSL	Spleen, tonsils, LNs	NSL	AX & Popliteal LNs
IMVRP-5	NSL	Spleen, tonsils, LNs	NSL	Popliteal LN
IMVRP-6	NSL	Spleen, tonsils, LNs	Rhinitis	Bone marrow
IMVRP-7	Perivascular cuffs	Spleen, tonsils, LNs	NSL	N/A
IMCTRL-1	Encephalitis	Spleen, tonsils, TB LN	Chronic rhinitis	Stomach
IMCTRL-2	NSL	Spleen, tonsils, LNs	NSL	N/A
IMCTRL-3	Meningo-encephalitis	Spleen, tonsils, LNs	NSL	Popliteal LN
IMCTRL-4	Encephalitis	Spleen, LNs	Pleural fibrosis	Stomach
IMCTRL-5	Encephalitis	Spleen, tonsils, LNs	NSL	N/A
IMCTRL-6	Encephalitis	Spleen, LNs	NSL	Tonsil, bone marrow
Intradermal Delivery of WEVEE VRP Vaccine (IDVRP) or Control (IDCTRL)				
IDVRP-1	NSL	Spleen, tonsil	NSL	PTGL, popliteal LN
IDVRP-2	Perivascular cuff	Spleen, tonsils, LNs	Gastritis	N/A
IDVRP-3	NSL	Spleen, tonsils, LNs	Gastritis	Popliteal LN
IDVRP-4	Perivascular cuff	Spleen, tonsils, LNs	NSL	PTGL, popliteal LN
IDVRP-5	NSL	Spleen, LNs	Gastroenteritis	Popliteal LN
IDVRP-6	NSL	Spleen, tonsils, LNs	Rhinitis, gastritis	N/A
IDVRP-7	Perivascular cuff	Spleen, tonsils, LNs	Pleural fibrosis	Popliteal LN
IDCTRL-1*	Meningo-encephalitis	Spleen	Seminal vesiculitis	Popliteal LN
IDCTRL-2	Encephalitis	Spleen, tonsils, LNs	Gastroenteritis	Popliteal LN
IDCTRL-3	Encephalitis, myelitis	Spleen, tonsils, LNs	NSL	Popliteal LN
IDCTRL-4	Encephalitis	Spleen, LNs	NSL	Tonsil
IDCTRL-5	Hydrocephalus, encephalitis, myelitis	Spleen, tonsils, LNs	NSL	Stomach

IDCTRL-6*	Meningo-encephalitis, spinal meningitis	Spleen, tonsils, LNs	NSL	Popliteal LN
Subcutaneous Delivery of WEVEE VRP Vaccine (SCVRP)				
SCVRP-1	NSL	Spleen, tonsils, LNs	NSL	Olfactory bulb
SCVRP-2	NSL	Spleen, tonsils, LNs	Pleural fibrosis	Duodenum
SCVRP-3	Perivascular cuff	Spleen, tonsils, MES LN	NSL	N/A
SCVRP-4	NSL	Spleen, tonsils, LNs	NSL	Popliteal LN
SCVRP-5	NSL	Spleen, tonsils, LNs	NSL	N/A
SCVRP-6	NSL	Spleen, tonsils, LNs	NSL	N/A
SCVRP-7	NSL	Spleen, tonsils, LNs	NSL	Popliteal LN

*euthanized on day 8 post-exposure (PE); all other NHPs were euthanized at study endpoint, day 28 PE
WEVEE = western, eastern, and Venezuelan equine encephalitis;
NSL = no significant lesion, LN = lymph node, TB = tracheobronchial, MES = mesenteric, AX = axillary, PTGL = pituitary gland, N/A = not applicable
